# Patient-reported outcomes after one year of periodontal treatment at public specialist dental clinics in Peninsular Malaysia

**DOI:** 10.1186/1471-2458-12-S2-A4

**Published:** 2012-11-27

**Authors:** Tuti Ningseh Mohd Dom, Syed Mohamed Al Junid, Mohd Rizal Abd Manaf, Khairiyah Abd Muttalib, Ahmad Sharifuddin Mohd Asari, Rasidah Ayob, Yuhaniz Ahmad Yaziz, Noorlin Ishak, Hanizah Abdul Aziz, Noordin Kasan

**Affiliations:** 1 Faculty of Dentistry, Universiti Kebangsaan Malaysia, Jalan Raja Muda Abdul Aziz, 53000 Kuala Lumpur, Malaysia; 2 United Nations University-International Institute for Global Health, Universiti Kebangsaan Malaysia Medical Centre, Jalan Yaacob Latiff, 56000 Kuala Lumpur, Malaysia; 3 Department of Community Health, Universiti Kebangsaan Malaysia Medical Centre, Jalan Yaacob Latiff, 56000 Kuala Lumpur, Malaysia; 4 Oral Health Division, Ministry of Health, Malaysia, 62590 Putra Jaya, Malaysia

## Background

Success of most periodontal treatment modalities as demonstrated by clinical parameters is well-established. These measurements, however, do not capture the full impact of treatment on the oral health status and health-related quality of life (QoL) of the patients. This study aims to assess the effectiveness of periodontal treatment provided at selected government specialist dental clinics using patient-reported outcomes.

## Materials and methods

This prospective study involved patients who visited five randomly selected government periodontics clinics in Malaysia for treatment of periodontitis. Following ethics approval, all patients meeting the selection criteria were recruited over a period of eight months and comprehensive periodontal treatment ensued for a period of twelve months. Diagnosis of periodontitis was based on presence of periodontal pockets at least 4mm deep. At the end of the study period periodontal treatment outcomes were assessed using two patient-centred outcome measures: Oral Health Impact Profile (OHIP-14S) and Euroqol EQ-5D-3L index.

## Results

A total of 145 patients started periodontal treatment but only 139 (95.8%) completed the 12-month study period. At post-treatment, patients had significantly improved oral health-related QoL (P<0.001) Wilcoxon Signed Rank Test; median OHIP-14S score of 7.0 at post-treatment vs 22.0 at baseline). Further, Cohen’s effect size was calculated to be 1.1, suggesting practical significance. Improvement in general health status was indicated by gain in EQ5D-utility and Visual Analogue scores at post-treatment (P<0.001 Wilcoxon Signed Rank Test; median utility score of 1.0 vs 0.73 and median VAS of 80 vs 70 at post-treatment and baseline respectively). Improvements in clinical parameters also reflected positive outcomes consistent with that of patient-reported outcomes.

## Conclusion

Periodontal treatment provided at the selected government periodontics clinics in Malaysia is effective in improving oral health status and health-related quality of life of the patients. Employing patient reported outcome measures in assessing effectiveness of dental health services will be an added value in providing high quality patient-centred healthcare.

